# Women in Leadership in State and Regional Orthopaedic Societies

**DOI:** 10.5435/JAAOSGlobal-D-21-00317

**Published:** 2022-04-05

**Authors:** Tolulope Ramos, Roxanne Daban, Nisha Kale, Symone Brown, Cadence Miskimin, Lisa K. Cannada, Mary K. Mulcahey

**Affiliations:** From the Tulane University School of Medicine, New Orleans, LA (Ramos, Daban, and Kale); the Department of Orthopaedic Surgery, Tulane University School of Medicine, New Orleans, LA (Brown, Miskimin, and Dr. Mulcahey); and the Novant Health, Jacksonville, FL (Dr. Cannada).

## Abstract

**Introduction:**

Female representation in orthopaedics remains low compared with other specialties. Recently, several studies have examined the membership composition and leadership roles of women in orthopaedic societies. However, there is a paucity of information on the possible connection between the number of women within state and regional orthopaedic societies and women who serve in leadership roles within these societies.

**Methods:**

Authors invited executive directors of 51 state and four regional orthopaedic societies to participate in an anonymous 14-question web-based survey about female members and women in leadership positions within these societies. The survey asked about female membership composition, the percentage of male and female practicing orthopaedic surgeons in the state/region (if available), and female representation on the Board of Directors of these societies. Data were analyzed for relationships between ordinal variables.

**Results:**

Forty-nine executive directors (89.1%) responded to the survey. Among respondents, there was a statistically significant positive correlation between the percentage of female members and women leaders (*P* = 0.015). Thirty-two executive directors (68.1%) reported between 1% and 10% female members, 7 (14.9%) had between 11% and 20% female members, and 2 (4.3%) had no female members. Twenty-five societies (52.1%) have never had a female president. Of 17 societies (32.7%) that had female presidents, 15 (75.0%) reported having just one female president. In addition, of these 17 societies, 12 (70.6%) reported having at least one acting female president within the past 10 years.

**Discussion:**

Our study demonstrates a positive correlation between female members and women on the Board of Directors in regional and state orthopaedic societies. Twelve societies had female presidents within the past 10 years. Female representation in leadership positions may help with the recruitment of female orthopaedic surgeons and improve diversity in orthopaedics. Future studies should evaluate different methods of increasing female membership and leadership positions in state and regional orthopaedic societies.

The presence of women in medicine has been increasing over the past 40 years; however, female representation in orthopaedic surgery remains low.^1,2^ In 2018, Chambers et al^2^ examined the prevalence of women in orthopaedic residency from 2005 to 2017, subspecialty societies in 2016, and research societies in 2016.^2^ Using the Association of American Medical Colleges and Accreditation Council for Graduation Medical Education databases as well as surveying members of the American Academy of Orthopaedic Surgeons (AAOS), the authors found that women represented approximately 14% of residents in orthopaedic surgery, 8.7% of orthopaedic surgery professors, and 6.5% of AAOS members.

Studies have examined factors that contribute to the low number of women in orthopaedics. In 2011, Baldwin et al^3^ found that having mentors of the same sex is an important factor in motivating women to pursue orthopaedics. Outreach programs such as the Perry Initiative and Nth dimension provide early exposure to encourage women to pursue orthopaedic surgery.^4^ Unfortunately, a 2019 study by Heimstra et al^5^ found that the lack of female role models in orthopaedic surgery further contributes to the perception that the field is not for women.

There continues to be a paucity of women who occupy leadership roles within orthopaedic surgery.^5^ In 2019, Saxena et al^6^ found a strong positive correlation (*r* = 0.2333; *P* = 0.0495) between the number of female members and the percentage of women on the Board of Directors in orthopaedic specialty societies. However, it is unclear whether a similar trend is present within state and regional orthopaedic societies. The purpose of this study was to determine whether a relationship exists between the percentage of women members within state and regional orthopaedic societies and the percentage of women occupying leadership positions within these societies.

## Methods

After obtaining approval from our Institutional Review Board, a 14-question anonymous survey was distributed to the executive directors of 51 state and four regional orthopaedic societies through the assistance of the AAOS Board of Councilors. Executive directors were invited to participate in the study through e-mail with a link to the survey and an explanation of the study aims. Follow-up emails were sent 2 and 4 weeks later by the Board of Councilors and authors to encourage additional participation.

The survey included questions about the state or regional society that participants represented, the percentage of female members in the state/regional society, the percentage of male and female practicing orthopaedic surgeons in the state/region, female representation on the Board of Directors of the state/regional society, and female membership in the state/regional society (Supplemental Appendix, http://links.lww.com/JG9/A212). The executive directors were asked to complete one survey per state or regional society that they represented. No identifying information was collected from the survey. Study data were collected and managed using SurveyMonkey.

Categorical thresholds were analyzed by examining distributions of the raw data between orthopaedic surgeons. Spearman correlation was used to analyze the relationship between ordinal variables. A *P* value of < 0.05 was considered statistically significant.

## Results

The survey was distributed to 51 executive directors of state societies and four executive directors of regional societies (N = 55) (Table 1), of whom 49 responded (response rate 89.1%). Within our sample, 46 respondents indicated that they represented a state society (94.0%). Three respondents (6%) represented a regional society, with one representative each from the Mid-America Orthopaedic Association, Western Orthopaedic Association, and Eastern Orthopaedic Association.

**Table 1:** 

Orthopaedic State Societies		
Alabama Orthopaedic Society Alaska State Orthopaedic Society Arizona Orthopaedic Society Arkansas Orthopaedic Society California Orthopaedic Association Colorado Orthopaedic Society Connecticut Orthopaedic Society Delaware Society of Orthopaedic Surgeons Florida Orthopaedic Society Georgia Orthopaedic Society Hawaii Orthopaedic Association Idaho Orthopaedic Society Illinois Association of Orthopaedic Surgeons Indiana Orthopaedic Society Iowa Orthopaedic Society Kansas Orthopaedic Society Kentucky Orthopaedic Society Louisiana Orthopaedic Association Maine Society of Orthopaedic Surgeons	Maryland Orthopaedic Association Massachusetts Orthopaedic Association Michigan Orthopaedic Society Minnesota Orthopaedic Society Mississippi Orthopaedic Society Missouri State Orthopaedic Association Montana Orthopaedic Society Nebraska Orthopaedic Society Nevada Orthopaedic Society New Hampshire Orthopaedic Society New Jersey Orthopaedic Society New Mexico Orthopaedic Association New York State Society of Orthopaedic Surgeons, Inc. North Carolina Orthopaedic Association North Dakota Orthopaedic Society	Ohio Orthopaedic Society Oklahoma State Orthopaedic Society Oregon Association of Orthopaedic Surgeons Pennsylvania Orthopaedic Society Puerto Rico Orthopaedic Society Rhode Island Orthopedic Society South Carolina Orthopaedic Association South Dakota Orthopaedic Society Tennessee Orthopaedic Society Texas Orthopaedic Association Utah State Orthopaedic Society Vermont Orthopaedic Society Virginia Orthopaedic Society Washington State Orthopaedic Association West Virginia Orthopaedic Society Wisconsin Orthopaedic Society Wyoming Orthopaedic Society

Orthopaedic Regional Societies		
Mid-America Orthopaedic Association Western Orthopaedic Association Eastern Orthopaedic Association Southern Orthopaedic Association

The study's survey was distributed to fifty-five orthopaedic state and regional societies listed in this table.

Thirty-two executive directors (68.1%) reported between 1% and 10% of their members were female, 7 (14.9%) had between 11% and 20% female members, and 2 (4.3%) had no female members. Sixteen executive directors (34.1%) reported having greater than 200 practicing male orthopaedic surgeons in their state/region, while in contrast 18 executive directors (45%) reported having less than 100 practicing female orthopaedic surgeons in their state/region.

Most of the respondents reported having greater than 10 orthopaedic surgeons on their Board of Directors (N = 21, 43.8%). Twenty-two (46.8%) reported no female members on their board, and 19 (40.4%) reported between 1 and 2 members of their board were female. A statistically significant, strong positive correlation was observed between the percentage of female members in a society and the number of female members on the Board of Directors (*r*_s_ [47] = 0.358, *P* = 0.015). A statistically moderate positive correlation was also observed between societies that had a higher number of orthopaedic surgeons on their Board of Directors and societies that had a high number of female members on their Board of Directors (*r*_s_ [47] = 0.486, *P* = 0.001).

**Figure 1:**
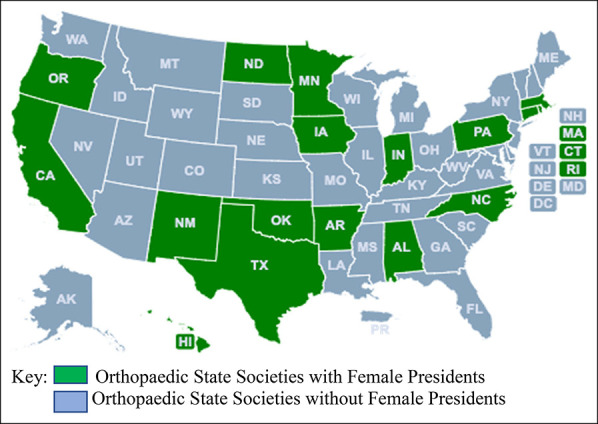
Map showing orthopaedic state societies with female presidents (N = 17).

**Figure 2:**
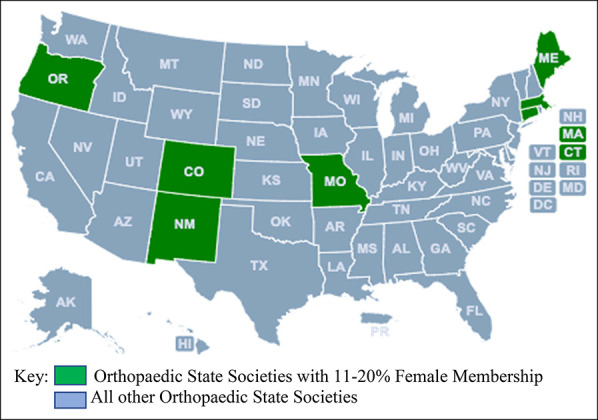
Map showing orthopaedic state societies with 11% to 20% female membership (N = 7).

More than half of the respondents reported that their state societies never had a female president (N = 25, 52.1%). For societies that did have female presidents (N = 17, 32.7%) (Figure 1), 15 (75.0%) reported having just one female president. Moreover, of these 17 societies, 12 (70.6%) reported having at least one female president within the past 10 years. Among the seven state societies that had between 11% and 20% female membership, 4 (57.1%) had a female president and 6 (85.7%) had female members on their Board of Directors (Figure 2). A weak positive correlation was observed between regional societies that had a female president and state/regions that had a large number of practicing female orthopaedic surgeons (*r*_s_ [38] = 0.340, *P* = 0.037). State societies that had a female president were also more likely to have female members on their Board of Directors (*r*_s_ [47] = 0.357, *P* = 0.014).

Most of the executive directors reported that their societies had either 3 to 4 membership categories (N = 23, 48.9%) or 1 to 2 membership categories (N = 18, 36.7%). Nineteen societies (41.3%) had less than 100 dues paying members of either sex. Seventeen executive directors (58.6%) reported less than 100 dues paying members were female, and 10 (34.5%) reported that less than 50 dues paying members were female. Societies with more dues paying memberships were more likely to have a large number of orthopaedic surgeons on their Board of Directors (*r*_s_ [46] = 0.665, *P* < 0.005), more membership categories (*r*_s_ [46] = 0.415, *P* = 0.004), and more practicing male orthopaedic surgeons in their state/region (*r*_s_ [45] = 0.477, *P* = 0.001).

## Discussion

Our study found that practicing female orthopaedic surgeons are markedly underrepresented in state and regional orthopaedic societies. In addition, our study demonstrated that few women comprise the Board of Directors of state and regional orthopaedic societies. However, we found a positive relationship between the number of female members in a society and the presence of female orthopaedic surgeons on the Board of Directors. Our study suggests that female leaders proportionately represent female orthopaedic members of state and regional societies. Although, female orthopaedic surgeons are overall underrepresented relative to the number of practicing orthopaedic surgeons in the general population that these societies represent. This finding is consistent with those of Saxena et al,^6^ who found that the presence of female leaders in orthopaedic specialty societies correlates with the number of female members.

Having diverse providers in the field of orthopaedic surgery may be beneficial for patient care. In 2011, Borkhoff et al^7^ found that women faced barriers such as minimization of their symptoms and attribution of symptoms to emotional rather than physical causes when interacting with their primary care physicians and orthopaedic surgeons throughout the referral and recommendation process for total joint arthroplasty. They suggested interventions such as cultural competency training to help improve patient-physician interaction and reduce gender disparity. Similar to other professions, male and female physicians display different communication styles that affects the patient-physician relationship.^8^ Female physicians are more likely to address a patient's psychosocial needs and emphasize preventive services than their male counterparts.^8,9^ Furthermore, patients of female physicians are more likely to display a higher level of satisfaction with their care.^8^

In 2019, Dineen et al reported that male and female patients appreciated traits such as the ability to sympathize and provide empathy in an orthopaedic surgeon. Furthermore, they reported that female patients showed a preference for an orthopaedic surgeon who inquired about patients' feelings and emotions.^9^ In 2009, Bertakis^8^ reported that female physicians displayed empathy toward patients by spending time learning about patients' feelings and discussing preventive care. These studies support the fact that female physicians possess specific attributes which may aid patient-centered care and strengthen the doctor-patient relationship.

Several pipeline programs, such as the Perry Initiative and Nth Dimension, have been created with the goal of encouraging women to enter the field of orthopaedic surgery.^10,11^ These programs provide mentorship for future female orthopaedic surgeons. According to Mulcahey et al, mentorship opportunities may be important in improving diversity in orthopaedics.^12,13^ Moreover, O'Connor^11^ found that having a same-sex mentor was considered important by 59% of the female medical students as opposed to 25% of the male medical students. Increasing the number of women in leadership positions within orthopaedic societies will help recruit more women into orthopaedics.

Although our study showed a correlation between the number of women on the Board of Directors and female members in state and regional orthopaedic societies, 15 (75%) societies that have had women on the Board of Directors have only had one female president. Moreover, we found that there was a weak positive correlation (*r*_s_ [38] = 0.340, *P* = 0.037) between societies that had a female president and states/regions that had a large number of practicing female orthopaedic surgeons.

The disparity between the number of male and female orthopaedic surgeons serving on the Board of Directors may be due to the large number of male members, as compared with female members in the societies. Only 7% of the orthopaedic surgeon members are women. It is expected that societies will have more male members. Attempts should be made to have women as representatives in visible roles in contributing to increasing diversity.

Although the numbers are still low in comparison to other specialties, female orthopaedic residents have increased from 0.6% of all orthopaedic residents in 1981 to 2001 to 14% in recent years.^1,2,14^ Because female orthopaedic surgery residents are young in their profession, they do not have the qualifications to be considered for leadership roles in state and local orthopaedic societies. Saxena et al^6^ noted that having seniority in an orthopaedic specialty society may play a role when becoming a member of that society's Board of Directors. In addition, Poon et al^15^ studied the presence of women in leadership positions of the Pediatric Orthopaedic Society of North America and found that there were less women in higher ranks of Pediatric Orthopaedic Society of North America, which they proposed was due to the time lag to advancement.

Our study also found that more female members were present in societies located in states/regions with overall more practicing orthopaedic surgeons. However, male orthopaedic surgeons comprised a larger portion of these societies. In addition, these societies had an overall larger number of orthopaedic surgeons on their Board of Directors. Saxena et al^6^ mentioned that “men can serve as allies to improve gender diversity.” With the large number of male practicing orthopaedic surgeons, there may be an opportunity for male leaders to participate in initiatives that could increase female representation in orthopaedics. For instance, male orthopaedic surgeons could become members of the Ruth Jackson Orthopaedic Society, which provides professional resources and mentorship to women in orthopaedics. State and regional societies should explore similar initiatives and involve male allies as sponsors who are in leadership positions. Moreover, a 2021 study by Chyu et al,^16^ which evaluated the current trends of women in urology leadership, noted that “promoting women in leadership could increase visibility of their practice patterns and translate to improved patient outcomes across the board.” Although the changes might take place over time, having more female leaders in state or regional societies can contribute different perspectives and resources that both male and female society members may bring back to their own practices.

There were several limitations to this study. First, the original survey had the lowest option of 100 to 200 members for any questions regarding membership. At 6 weeks, the survey was modified because of feedback from several participants that their societies had less than 100 female members. The options of “<100” and “Other (please specify)” were added to all relevant questions. Because of SurveyMonkey's limitations of repeated survey submissions, a word document of the survey was sent to the societies after the new options were added. Second, we used larger scales in our study that made stratification challenging. The highest choice for female membership chosen by only seven participants was 11% to 20%, while most societies reported having zero or 1% to 10% female membership. Future studies should consider using smaller scales to accurately reflect female membership. Future studies should further stratify using smaller scales that are reflective of the national trend of female orthopaedic surgeons. As mentioned in the Introduction, AAOS is comprised of 6.5% female members. The low general population of female orthopaedic surgeons can affect the overall female membership composition of state and regional societies. Future studies should include survey choices that are 6.5% or less. Third, our study revealed that 12 of the 17 societies with female presidents reported having at least one female president within the past 10 years. However, it does not address changes in gender composition of these societies over time. Collection of more longitudinal data may have been beneficial for assessing relevant patterns and trends. We also did not compare the average number of years in practice between female and male society members. The limited number of women in leadership roles could be attributed to practicing in the profession for a shorter amount of time compared with their male counterparts. Future studies should study the average number of years of practice between male and female society members and leaders. Finally, our study did not consider state population totals, which may affect the number of practicing orthopaedic surgeons within the state. Future studies should evaluate the relationship of state populations and population density, total number of practicing orthopaedic surgeons, and the percentage of practicing female orthopaedic surgeons.

## Conclusion

Our study demonstrated a positive correlation between the number of female members and women on the Board of Directors in state and regional orthopaedic societies. This finding is consistent with previous research demonstrating that female mentors encourage more women to join the field of orthopaedics. However, only a few societies had female presidents within the past 10 years. Increased female representation in orthopaedics could provide diverse perspectives, which may enhance patient care. Future studies should seek to identify and implement effective ways to increase gender diversity and women in leadership positions within the field of orthopaedic surgery.
